# Latent class analysis identifies distinct pain phenotypes in newly diagnosed systemic juvenile idiopathic arthritis

**DOI:** 10.1186/s13075-025-03534-7

**Published:** 2025-03-31

**Authors:** Hui Zhang, Xiaoqiong Wei, Wei Liu, Hongyao Leng, Qiao Shen, Xin Wan, Ximing Xu, Xianlan Zheng

**Affiliations:** 1 Department of Nursing, National Clinical Research Center for Child Health and Disorders, Ministry of Education Key Laboratory of Child Development and Disorders, Chongqing Key Laboratory of Pediatrics, Children’s Hospital of Chongqing Medical University, Chongqing, China; 2 Department of Rheumatology and Immunology, Children’s Hospital of Chongqing Medical University, Chongqing, China; 3 Department of Anesthesiology, Children’s Hospital of Chongqing Medical University, Chongqing, China; 4 Big Data Center for Children’s Medical Care, Children’s Hospital of Chongqing Medical University, Chongqing, China

**Keywords:** Systemic juvenile idiopathic arthritis, Pain phenotype, Latent class analysis, IL-10, Electronic health records

## Abstract

**Background:**

Patients with systemic juvenile idiopathic arthritis (sJIA) exhibit highly heterogeneous pain manifestations, which significantly impact their quality of life and disease prognosis. An understanding of the pain phenotypes for this disorder and their influencing factors is crucial for individualized pain management.

**Objective:**

To explore the pain phenotypes of newly diagnosed sJIA patients via latent class analysis (LCA), analyse the influencing factors of these phenotypes, and evaluate the impacts of different pain phenotypes on short-term inpatient outcomes.

**Methods:**

A retrospective cohort study was conducted by collecting the electronic health records of 165 patients who were first diagnosed with sJIA at the Children’s Hospital of Chongqing Medical University from January 2018 to July 2024. Patient pain characteristics, laboratory indicators, and inpatient outcome data were extracted. LCA was used to identify pain phenotypes, and multivariate logistic regression was used to analyse the influencing factors. The Lanza–Tan–Bray method and the data combination analysis technique were applied to evaluate the relationships between pain phenotypes and clinical outcomes.

**Results:**

LCA categorized the pain phenotypes of sJIA patients into three distinct classes, including (1) Class 1: inflammation-related moderate to severe pain with functional impairment (53.9% of patients); (2) Class 2: mild intermittent pain with extra-articular symptoms (19.4% of patients); and (3) Class 3: no joint pain with mild functional impairment (26.7% of patients). The analysis revealed that age (*P* = 0.023) and serum IL-10 levels (*P* = 0.047) were significant factors influencing pain phenotypes. Significant differences were observed among different pain phenotypes in terms of hospital stay duration, intrahospital department transfer rates, and pain status at discharge.

**Conclusion:**

Pain in sJIA patients can be classified into three distinct phenotypes, which are influenced by factors such as age and IL-10 levels. The identification of these pain phenotypes has important clinical significance for developing individualized pain management strategies.

**Supplementary Information:**

The online version contains supplementary material available at 10.1186/s13075-025-03534-7.

## Introduction

Juvenile idiopathic arthritis (JIA) is one of the most common chronic rheumatic diseases occurring in childhood, and it affects approximately 1 in every 1,000 children worldwide [1, 2]. Systemic juvenile idiopathic arthritis (sJIA) accounts for approximately 10% of JIA cases and is characterized by prominent systemic inflammatory responses, including recurrent fever, joint pain, and multisystem involvement [3]. Additionally, sJIA is characterized by inappropriate activation of the innate immune system and excessive secretion of proinflammatory cytokines such as interleukin-1 (IL-1), IL-6, and IL-18 [4]. Within only two years of onset, joint ankylosis can develop, thereby indicating that sJIA is one of the primary subtypes of JIA that is associated with joint disability [5].

Pain is one of the most commonly reported and significant symptoms in patients with sJIA and is a leading cause of disability [6]. Pain has a profound negative impact on patients’ quality of life [7], disease prognosis, and mental health, thus leading to limitations in daily activities and affecting school attendance and social interactions [8, 9]. In sJIA patients, pain manifestations are complex and diverse, whereby they encompass not only joint pain but also systemic pain caused by widespread inflammatory responses [3]. However, in clinical practice, pain symptoms are often overlooked, with more attention typically devoted to inflammation and functional impairment [10]. The heterogeneity of pain in sJIA patients is reflected in multiple aspects, including the location, intensity, and duration of pain, as well as accompanying symptoms that vary among individuals [11, 12]. Some patients respond well to standard pain treatments, whereas others find it difficult to achieve satisfactory relief despite multiple interventions [13]. Approximately 20–30% of sJIA patients continue to experience varying degrees of pain even after treatment with biologics [14]; this scenario is considered to represent chronic pathological pain that biologics cannot fully control. As the disease progresses, the pain associated with sJIA may become more diverse, which is potentially related to noninflammatory pain mechanisms such as central sensitization, dysfunction of endogenous pain modulation systems, and psychological factors [15, 16].

Pain phenotypes refer to the specific pain characteristics and symptom patterns exhibited by patients with a particular disease, including the nature, location, and intensity of pain, as well as accompanying symptoms and responses to treatment [17, 18]. In recent years, research on pain phenotypes has received widespread attention in the management of chronic pain in adults, especially in fields such as rheumatic diseases and osteoarthritis [19]. Electronic health records (EHRs) provide extensive structured and unstructured data supporting clinical decision-making, and these records can encompass pain-related information across all stages of JIA diagnosis and treatment [20]. Moreover, EHRs can integrate patient-reported symptoms, physical examination findings, inflammatory markers, and imaging studies to objectively assess multidimensional pain data. Additionally, latent class analysis (LCA) [21], which is a statistical method for inferring latent categories or groups based on observed data, can help to reveal potential differences among patient populations with chronic pain and inflammatory diseases [22].

Professional organizations such as the Australian Paediatric Rheumatology Group [23], the British Society for Rheumatology [24] and the Japanese JIA Clinical Practice Guidelines [25] emphasize the importance of the early monitoring of pain in JIA patients for timely intervention. In the early onset and acute phases of sJIA, pain is mainly inflammatory in nature, whereby it arises from excessive activation of the immune system and the overexpression of cytokines [26]. Given the complexity and individual variability of pain in sJIA patients, the identification of these different pain categories and their early characteristics is crucial for optimizing patient management and improving long-term health outcomes.

This study utilized EHRs and LCA to investigate pain phenotypes and their influencing factors in newly diagnosed and early-stage sJIA patients. This study aimed to comprehensively describe pain characteristics, identify key factors influencing different pain phenotypes, and evaluate the impacts of these pain phenotypes on short-term clinical outcomes. Furthermore, this study explored the differences in inflammatory factors among different pain phenotypes in sJIA patients to reveal potential mechanisms of pain occurrence, thereby providing scientific evidence for early diagnosis and individualized pain management of this disease.

## Methods

### Patients and settings

This observational retrospective cohort study was conducted at the Children’s Hospital of Chongqing Medical University. We collected data from patients diagnosed with sJIA at our Yuzhong and Liangjiang campuses from January 2018 to July 2024 to construct the research cohort. Hospitalized cases were screened via the electronic medical record system, and ICD codes were manually reviewed to exclude cases with only a single suspected diagnosis without confirmation.

The inclusion criteria were as follows: (1) children who were first diagnosed with systemic sJIA between January 2018 and July 2024; (2) those who visited our hospital within two months after an initial out-of-hospital diagnosis and had detailed records of early pain symptoms and laboratory test results; and (3) ages ranging from 3 to 18 years. The exclusion criteria were as follows: (1) patients with other types of JIA; (2) those diagnosed with sJIA outside of the hospital with a diagnosis time exceeding two months; (3) patients with malignant diseases, immunodeficiency diseases, or other rheumatic autoimmune diseases; (4) patients with macrophage activation syndrome; and (5) patients with missing clinical data or laboratory examination results. This study was approved by the Ethics Committee of the Children’s Hospital of Chongqing Medical University and strictly adhered to the principles of subject privacy protection.

### Variables and data processing

#### General patient information and disease-related data

We collected data on patients’ sex, age, residential region classification, mode of birth, feeding method, family history of rheumatic or autoimmune diseases, time of first diagnosis, comorbid diagnoses, number of outpatient visits outside of the hospital and time of initial symptom onset.

#### Pain assessment and classification

Based on the Outcome Measures in Rheumatology (OMERACT) [27, 28] pain assessment framework and the American College of Rheumatology (ACR) [29] guidelines on JIA, combined with the routine processes of inpatient inquiries and physical examinations in our hospital, we extracted descriptions of patients’ pain symptoms from four sections of the inpatient medical records: “Chief Complaint,” “Current Medical History,” “Physical Examination,” and “Attending Physician Ward Round Records.” The evaluation of JIA pain characteristics was divided into the following seven factors.

First, we collected data on the temporal characteristics of the child’s pain, including persistent, intermittent, and paroxysmal pain. Second, we recorded whether pain was the initial clinical manifestation of the disease. Additionally, we documented the joint locations of pain by using the Juvenile Arthritis Disease Activity Scale (JADAS) 27-joint version [30]. The JADAS-27 is primarily used to evaluate 27 joints throughout the body, including the cervical spine, bilateral elbows, bilateral wrists, first to third metacarpophalangeal joints, 10 proximal interphalangeal joints, bilateral hips, bilateral knees, and bilateral ankles, thereby providing a comprehensive assessment of joint involvement in sJIA. Moreover, this version has been widely employed in paediatric rheumatology research in China [31].

We assessed pain intensity by using the Verbal Rating Scale (VRS) for children [32], and we categorized pain intensity into no pain, mild pain, moderate pain, and severe pain categories. Studies have shown that the VRS scale demonstrates high reliability in adolescents and good discriminatory validity for the assessment of different types of pain [33]. To better reflect the clinical reality and flexibly capture the children’s pain complaints, we used a revised verbal rating standard to classify and define pain intensity as follows. Mild pain: the child feels pain but can tolerate it and describes it as “mild pain,” “not severe,” or “bearable.” Moreover, daily life is normal, sleep is unaffected, and no analgesics are needed (or only minor interventions are needed). Moderate pain: pain exerts a certain impact on daily activities and is described as “obvious pain”, “moderate pain” or “relatively obvious pain.” Additionally, the child experiences discomfort but can still tolerate it; moreover, analgesic medication or nonpharmacological interventions are usually needed before admission or during hospitalization. Severe pain: the pain is intense and intolerable for the child and is described as “severe pain” or “unbearable.” Analgesic medication is needed, and the pain cannot be completely relieved; in addition, sleep is disturbed, and the child may be forced to adopt specific postures or may be accompanied by autonomic nervous system disorders. The pain intensity was evaluated by two independent clinicians based on the medical records; moreover, only patients with fully consistent assessments were retained, and those with discrepancies or missing pain descriptions were excluded. Additionally, we recorded extra-articular pain sites, including headache, abdominal pain, chest pain, and muscle pain; evaluated joint swelling and skin temperature elevation over the joint surface via physical examination to assess inflammatory pain characteristics; and assessed functional impairment, including limited joint mobility and limping.

#### Laboratory assessments

The laboratory test data that were collected within 24 h after patient admission included C-reactive protein (CRP), the erythrocyte sedimentation rate (ESR), and procalcitonin, TNF-α, IL-6, and IL-10 levels. If samples could not be immediately collected, the first set of data obtained during hospitalization were used.

#### Outcome variables

The following primary outcome variables were utilized in this study. (1) Length of hospital stay. (2) Internal transfer during hospitalization, which referred to whether the patient was transferred between departments during their hospital stay (yes/no). (3) Pain status at discharge, which was classified according to the discharge records of the patients and was divided into the following two categories: no pain symptoms and persistent pain. Persistent pain indicates that the pain has not been completely alleviated.

### Statistical analysis

Statistical analysis was performed using SPSS 25.0 and MPLUS 7.4.0 software. First, descriptive statistical analysis was conducted on the data. LCA identified potential pain phenotypes in systemic JIA patients. Models were evaluated using AIC, BIC, and aBIC methods (smaller values indicate better fit) and entropy values (0–1; higher values indicate better classification accuracy). Likelihood ratio tests (Lo–Mendell–Rubin and bootstrap-based tests) were used to compare model performance; a p value < 0.05 indicated that the k-class model was significantly better than the k-1 class model. For the univariate analysis, the normality of the continuous variables was tested using the Shapiro–Wilk test. Normally distributed variables were analysed via one-way ANOVA; additionally, nonnormally distributed variables were analysed via nonparametric tests (such as the Kruskal–Wallis test). Categorical variables were analysed using chi-square tests with Bonferroni correction for pairwise comparisons. Multivariate logistic regression was used to analyse the factors influencing latent pain categories. Additionally, the Lanza–Tan–Bray (LTB) [34] method was employed for regression mixture model analysis to explore the relationships between discharge pain status, in-hospital department transfers, and latent categories. The Bolck–Croon–Hagenaars (BCH) method was used to examine the associations between latent categories and lengths of hospital stay, including pairwise comparisons.

## Results

### Demographic characteristics of the study subjects

This study included 165 patients with sJIA. The average age of the patients was 8.8 ± 3.6 years (ranging from 3.0 to 15.9 years). A detailed summary of the patients’ demographic profiles is presented in Table 1.

**Table 1:** Demographic characteristics of sJIA patients (*N* = 165)

Variable	Value
**Age**, *n***(%)**	
3 ≤ Age < 6 years	50 (30.3)
6 ≤ Age < 9 years	32 (19.4)
9 ≤ Age < 12 years	46 (27.9)
Age ≥ 12 years	37 (22.4)
**Gender**,** n (%)**	
Male	94 (57.0)
Female	71 (43.0)
**Residence**,** n (%)**	
Urban	71 (43.0)
Town	34 (20.6)
Rural	60 (36.4)
**Feeding Method**,** n (%)**	
Breastfeeding	129 (78.2)
Formula	13 (7.9)
Mixed	19 (11.5)
Unspecified	4 (2.4)
**Mode of Delivery**,** n (%)**	
Vaginal	77 (46.7)
Caesarean	77 (46.7)
Unknown	11 (6.6)
**Family History of Rheumatic Disease**,** n (%)**	
Yes	7 (4.2)
No	158 (95.8)
**Ethnicity**,** n (%)**	
Han	140 (84.85)
Tujia	8 (4.85)
Miao	4 (2.42)
Gelao	2 (1.21)
Yi	2 (1.21)
Others (Zhuang, Tibetan, Bouyei, Li, Dong, Zang)	6 (3.64)
Unspecified	4 (2.42)

### Latent class analysis and model fit

Models with one to five classes were assessed (Table 2). AIC and aBIC values decreased with greater class ranks, whereby they reached their lowest values in the three-class model, thus indicating a better fit, whereas the BIC favoured the two-class model. The two-class model exhibited the highest entropy (1.000), thereby reflecting very high classification accuracy, compared with the three-class model entropy value of 0.887. Significance tests revealed that increasing from one to two classes significantly improved the model (LMR and BLRT *p* < 0.001); however, when increasing from two to three classes, only the BLRT remained significant (*p* < 0.001), with the LMR being nonsignificant (*p* = 0.097), thus suggesting no significant improvement. The addition of more classes did not enhance the model fit. The three-class model exhibited balanced class proportions (0.539, 0.194, and 0.267), thereby avoiding small class sizes. When considering fit indices, classification accuracy, significance tests, and balanced class distributions, the three-class model was selected as the optimal model, despite the advantages of the two-class model in terms of the BIC and classification accuracy.

**Table 2:** Fit indices for latent class models of pain phenotypes in systemic juvenile idiopathic arthritis patients

Latent Class	AIC	BIC	aBIC	Entropy	LMR (*p*)	BLRT (*p*)	Category Probability
Class 1	2250.237	2293.720	2249.396	-	-	-	1.000
Class 2	1894.733	1984.806	1892.992	1.000	< 0.001	< 0.001	0.267, 0.733
Class 3	1867.584	2004.245	1864.941	0.887	0.097	< 0.001	0.539, 0.194, 0.267
Class 4	1869.752	2053.003	1866.209	0.984	0.317	0.333	0.444, 0.170, 0.267, 0.120
Class 5	1877.003	2106.843	1872.559	0.933	0.934	0.667	0.145, 0.284, 0.139, 0.163, 0.267

AIC: Akaike information criterion; BIC: Bayesian information criterion; aBIC: adjusted Bayesian information criterion; LMR: Lo–Mendell–Rubin likelihood ratio test; BLRT: bootstrap likelihood ratio test

### Identification and naming of latent classes

**Fig. 1:**
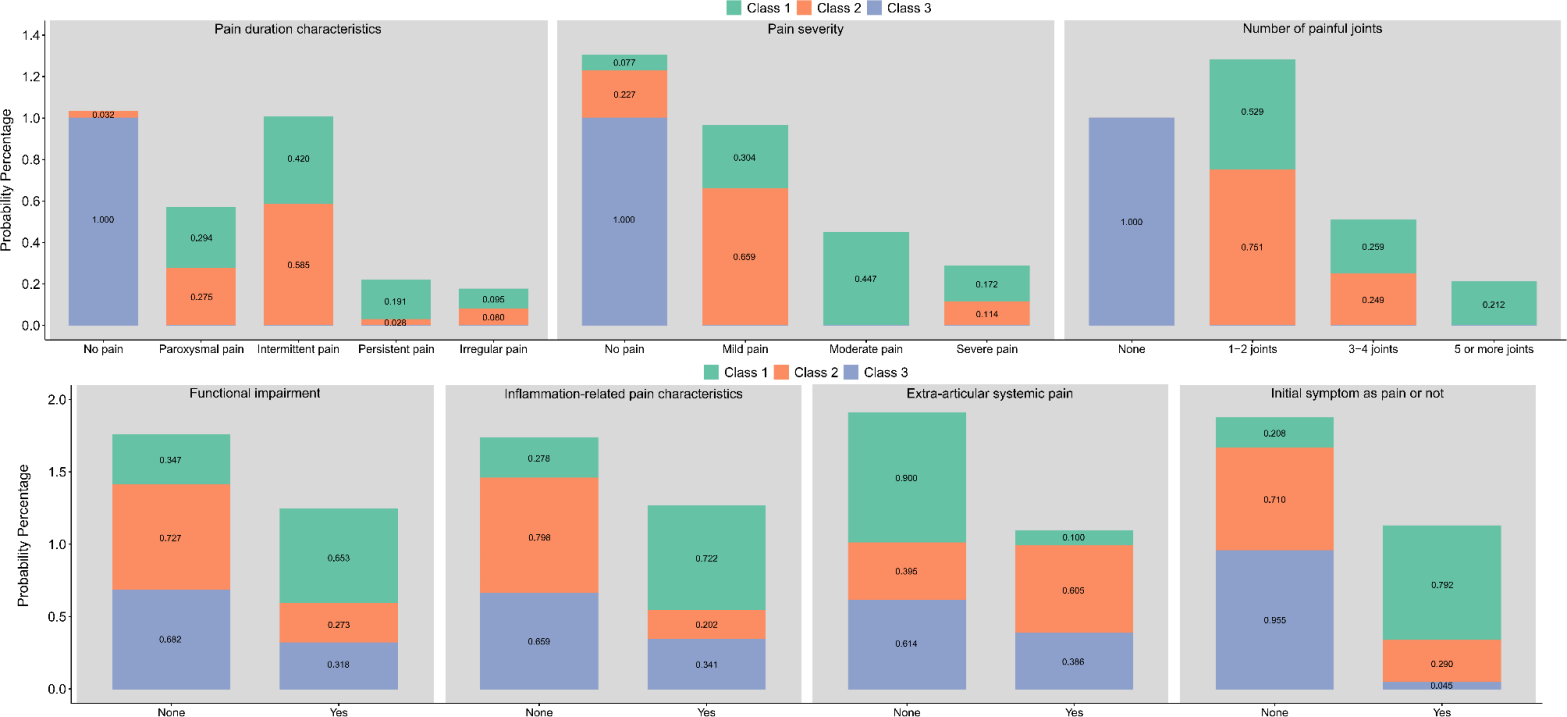
Conditional probabilities of latent pain phenotype categories in systemic juvenile idiopathic arthritis patients

The conditional probabilities of the best-fitting three-class model are shown in Fig. 1. Class 1 (C1): patients in this class experienced pain and functional impairment early in the disease, with certain inflammation-related pain characteristics being demonstrated. Based on these features, this class was termed the “Moderate-to-Severe Inflammation-Related Pain with Functional Impairment” type, which accounted for approximately 53.9% of the cases. Class 2 (C2): patients in this class primarily exhibited intermittent and mild pain, with pain being localized to one or two joints and minimal functional impairment being observed. However, many patients in this class also exhibited extra-articular, systemic pain symptoms (60.5%). Therefore, this class was termed the “Mild Intermittent Pain with Extra-Articular Symptoms” type, which represented approximately 19.4% of the cases. Class 3 (C3): patients in this class exhibited minimal overall symptoms, with almost no pain and no joint pain being observed in any of the patients. A small number of patients exhibited mild functional impairment and extra-articular symptoms. Thus, this class was termed the “No Joint Pain with Mild Functional Impairment” type, which accounted for approximately 26.7% of the cases.

### Factors influencing latent pain phenotypes in sJIA patients

Univariate analysis revealed significant differences among systemic JIA patients in terms of age, time from symptom onset to consultation, and blood IL-10 levels, whereas other characteristics, such as sex, residence, and family history, demonstrated no significant differences (see Table 3). A multivariate logistic regression further revealed that higher IL-10 levels significantly increased the odds of being classified in the C2 group versus the C1 group, thus indicating a strong association with this pain phenotype (Table 4). Additionally, younger age was a significant predictor for classification in the C3 group compared with the C1 group (*P* = 0.023), thereby suggesting that age plays a crucial role in differentiating these patient groups.

**Table 3:** Comparison of clinical and laboratory characteristics among different pain phenotypes in patients with systemic juvenile idiopathic arthritis

Variable		C1	C2	C3	χ² or F	*P*
Gender	Female	38 (42.7%)	12 (37.5%)	21 (47.7%)	0.799^b^	0.671
	Male	51 (57.3%)	20 (62.5%)	23 (52.3%)		
Age		9.88 (6.13 ~ 12.28)	9.63 (5.52 ~ 11.76)	7.75 (4.14 ~ 11.05)	**6.678** ^**a**^	**0.035**
Residence	Urban	38 (42.7%)	11 (34.4%)	20 (45.5%)	1.942^b^	0.746
	Town	18 (20.2%)	7 (21.9%)	11 (25.0%)		
	Village	33 (37.1%)	14 (43.8%)	13 (29.5%)		
Ethnicity	Han Chinese	75(84.3)	25(78.1)	40(90.9)	2.406	0.300
	Other Ethnic Minorities	14(15.7)	7(21.9)	4(9.1)		
Birth Method	Vaginal	44 (49.4%)	12 (37.5%)	21 (47.7%)	2.233^c^	0.708
	Caesarean	40 (44.9%)	18 (56.3%)	19 (43.29%)		
	Unknown	5 (5.6%)	2 (6.3%)	4 (9.1%)		
Feeding Method	Breastfeeding	73 (82.0%)	21 (65.6%)	35 (79.5%)	5.441^c^	0.455
	Formula	7 (7.9%)	3 (9.4%)	3 (6.8%)		
	Mixed	7 (7.9%)	7 (21.9%)	5 (11.4%)		
	Unknown	2 (2.2%)	1 (3.1%)	1 (2.3%)		
Family History of Rheumatic Diseases	No	85 (95.5%)	31 (96.9%)	42 (95.5%)	0.221^c^	1.000
	Yes	4 (4.5%)	1 (3.1%)	2 (4.5%)		
Number of Outpatient Hospitals Visited	0	16 (18.0%)	2 (6.3%)	7 (15.9%)	3.848^c^	0.704
	1	49 (55.1%)	20 (62.5%)	28 (63.6%)		
	2	20 (22.5%)	9 (28.1%)	8 (18.2%)		
	3	4 (4.5%)	1 (3.1%)	1 (2.3%)		
Time from Symptom Onset to Visit (Days)		20.00 (11.50 ~ 30.00)	14.00 (10.00 ~ 20.75)	14.50(7.00 ~ 25.00)	**6.212** ^**a**^	**0.045**
PCT		0.38 (0.12 ~ 1.25)	0.50 (0.23 ~ 1.25)	0.52 (0.15 ~ 1.24)	2.123^**a**^	0.346
ESR		58.00 (29.00 ~ 85.50)	64.00 (42.25 ~ 86.75)	75.00(42.50 ~ 98.50)	4.002^**a**^	0.135
CRP		68.40 (12.07 ~ 116.50)	57.95(18.33 ~ 121.75)	66.20(24.52 ~ 108.00)	0.626^**a**^	0.731
IL-10		4.16 (1.59 ~ 8.08)	8.16(3.94 ~ 10.17)	8.05(3.30 ~ 8.15)	**17.292** ^**a**^	**0.000**
IL-6		44.57 (15.67 ~ 103.67)	40.54 (29.35 ~ 55.48)	45.36 (22.84 ~ 82.40)	0.686 ^**a**^	0.710
TNF-α		0.80 (0.18 ~ 1.35)	1.23 (0.66 ~ 2.83)	1.23 (0.04 ~ 2.49)	5.731 ^**a**^	0.057

CRP: C-reactive protein; TNFα: tumour necrosis factor alpha; IL-10: interleukin-10; ESR: erythrocyte sedimentation rate; PCT: procalcitonin; *P* < 0.05: the result was statistically significant. Superscripts are defined as follows: a: Kruskal–Wallis test; b: chi-square test; c: Fisher’s exact test

**Table 4:** Analysis of latent pain phenotype categories using multivariate logistic regression in systemic juvenile idiopathic arthritis

Groups	B	SE	Wald	*p*	OR	95% CI
**C2 vs. C1**						
Constant	-1.352	0.648	4.359	0.037	-	-
Age	0.005	0.060	0.006	0.940	1.005	0.893 ~ 1.130
Time from Symptom Onset to Visit (Days)	-0.003	0.003	0.646	0.422	0.997	0.991 ~ 1.004
IL-10	0.054	0.027	3.934	**0.047**	1.055	1.001 ~ 1.113
***C3 vs. C1***						
Constant	0.012	0.525	0.000	0.982	-	-
Age	-0.125	0.055	5.172	**0.023**	0.882	0.792 ~ 0.983
Time from Symptom Onset to Visit (Days)	0.001	0.001	1.271	0.260	1.001	1.000 ~ 1.002
IL-10	0.042	0.027	2.398	0.121	1.043	0.989 ~ 1.099
***C2 vs. C3***						
Constant	1.364	0.672	4.123	0.042	-	-
Age	-0.130	0.069	3.522	0.061	0.878	0.767 ~ 1.006
Time from Symptom Onset to Visit (Days)	0.003	0.003	0.959	0.327	1.003	0.997 ~ 1.010
IL-10	-0.012	0.013	0.870	0.351	0.988	0.963 ~ 1.013

IL-10: interleukin 10; C1: class 1; C2: class 2; C3: class 3; SE: standard error; OR: odds ratio; 95% CI: 95% confidence interval

### Short-Term inpatient outcomes in sJIA patients based on pain phenotypes

Significant differences were observed in short-term inpatient outcomes among sJIA patients with different pain phenotypes, specifically with respect to internal transfers, pain status at discharge, and length of hospital stay (Table 5). Pairwise comparisons revealed that the proportion of patients without internal transfers was significantly greater in the C1 group than in the C2 group (χ² = 5.958, *p* = 0.015) and the C3 group (χ² = 24.721, *p* < 0.001). Similarly, the proportion of pain-free patients at discharge was significantly lower in the C1 group than in the C2 group (χ² = 7.502, *p* = 0.006) and the C3 group (χ² = 25.906, *p* < 0.001). The mean hospital stay was significantly shorter in the C1 group than in the C2 group (χ² = 7.094, *p* = 0.008) and the C3 group (χ² = 22.965, *p* < 0.001). No significant differences were observed between the C2 and C3 groups regarding these comparisons.

**Table 5:** Comparison of Short-Term hospitalization outcomes among different pain phenotypes of systemic juvenile idiopathic arthritis patients

Variable	Prob/Mean ± S.E.			χ²	*P*
	**C1 C2 C3**				
Internal Transfers				27.532	< 0.001
None	0.891	0.403	0.477		
Yes	0.109	0.597	0.523		
Discharge Pain Situation					
No pain	0.393	0.697	0.818	26.007	< 0.001
Persistent Pain	0.607	0.303	0.182		
Hospitalization Days	10.943 ± 0.592	15.225 ± 1.392	17.091 ± 1.138	25.375	< 0.001

C1: class 1; C2: class 2; C3: class 3

## Discussion

This is the first study to utilize LCA to classify pain phenotypes in 165 newly diagnosed patients with sJIA. By integrating clinical covariates such as pain intensity, temporal characteristics, pain location, degree of functional impairment, and accompanying symptoms, we identified three distinct pain phenotypes. These findings are consistent with results from other pain phenotype studies and emphasize the heterogeneity of pain in patients with arthritis [35, 36].

Patients in the C1 group exhibited moderate-to-severe persistent joint pain early in the disease course, which was accompanied by significant functional impairment that affected daily activities. Joint swelling and increased skin temperature suggest active intra-articular inflammation. These patients require aggressive pain management [37], including pharmacological interventions and nonpharmacological therapies such as physical therapy and rehabilitation training [38], in order to alleviate inflammation, reduce pain, and prevent joint destruction and functional loss. Early intervention is crucial for improving the long-term prognosis of these patients [39]. In the C2 group, patients primarily exhibited intermittent mild pain involving one or two joints, with relatively mild functional impairment being observed. However, 60.5% of these patients reported extra-articular systemic pain symptoms, such as muscle pain and abdominal pain. Previous studies have demonstrated that pain in sJIA patients is not only related to joint inflammation but may also be influenced by systemic inflammatory responses [40], thereby leading to diverse pain locations. Intermittent pain synchronizes with fever cycles, thus suggesting that systemic inflammation plays a significant role in pain exacerbation. From a clinical perspective, it is essential to closely monitor systemic symptoms and laboratory indicators to prevent the occurrence of serious complications, such as macrophage activation syndrome [41]. Treatment strategies should focus on both joint symptoms and systemic inflammation to prevent the occurrence of potential complications. Patients in the C3 group did not experience joint pain; however, these types of patients may exhibit mild functional impairment and other extra-articular pain symptoms, such as muscle pain, abdominal pain, and chest pain [42]. Additionally, some patients may develop joint pain only after systemic symptoms have subsided [2]. Continuous monitoring of disease progression and pain status in these patients is necessary to adjust treatment plans in a timely manner.

The results of multivariate logistic analysis revealed that age had a significant effect on different pain phenotypes. Patients categorized as C3 (“No Joint Pain with Mild Functional Impairment”) tended to be younger. This finding can be explained by the epidemiological characteristics of sJIA; specifically, younger children may exhibit different immune responses to sJIA inflammation, whereby they may primarily present with systemic symptoms such as high fever and rash while exhibiting less pronounced joint symptoms [43]. Studies have indicated that younger sJIA patients may exhibit distinct cytokine profiles [44], thus resulting in significant systemic inflammation but milder joint symptoms. Additionally, younger children may struggle to express or pinpoint the location of pain, thereby potentially leading to the underreporting of pain symptoms. An understanding of these age-related differences is crucial for timely diagnosis and management [45].

Compared with C1 patients, C2 patients exhibited higher IL-10 levels (*P* = 0.047), with each unit increase in IL-10 increasing the likelihood of being classified as C2 by 5.5%. C1 patients exhibited persistent severe joint pain and significant functional impairment, accompanied by joint swelling and increased skin temperature, thus indicating active intra-articular inflammation. IL-10 is a key anti-inflammatory cytokine that inhibits proinflammatory cytokines such as IL-1β, TNF-α, and IL-6 [46], thereby limiting inflammatory responses. Insufficient IL-10 levels can lead to elevated levels of inflammatory mediators [47], thereby activating nociceptors and lowering pain thresholds, which subsequently intensifies inflammation-associated pain. Additionally, low IL-10 levels may enhance neuronal excitability, thus amplifying pain perception. Conversely, elevated IL-10 levels modulate joint inflammation and alleviate pain and swelling. In neuropathic pain models, IL-10 reduces macrophage infiltration and TNF-α levels at nerve injury sites, thereby effectively mitigating pain. Furthermore, IL-10 promotes M2 macrophages and regulatory T-cell activity, thus regulating immune homeostasis [48].

These findings suggest that IL-10 plays a significant role in modulating pain and inflammation in sJIA patients [49]. Clinically, the enhancement of IL-10 activity may alleviate inflammatory and neuropathic pain by addressing immune dysregulation and neuronal hyperexcitability. Additionally, the IL-10 level may serve as a biomarker to identify patients with severe inflammatory pain phenotypes, thereby enabling the development of personalized pain management strategies. However, further research is needed to confirm the efficacy and safety of targeting IL-10 in clinical settings. The investigation of early pain phenotypes and inflammatory factors can help in elucidating the pathological mechanisms of this disease, thus providing new perspectives and data support for both basic and clinical research.

This study also revealed significant differences in the rates of internal department transfers, pain status at discharge, and length of hospital stay among sJIA patients with different pain phenotypes. C1 patients exhibited the lowest rate of internal transfers, whereas C2 and C3 patients exhibited comparatively higher rates. This observation may be due to the fact that patients in Classes 2 and 3 exhibit more complex clinical presentations, with atypical initial symptoms requiring multidisciplinary consultations and investigations [50, 51], thus leading to increased internal transfer rates. Additionally, the average length of hospital stay for C1 patients was significantly shorter than the average lengths of stay for C2 and C3 patients. The possible reason for this result is that C1 patients exhibit clear pain symptoms, and the diagnostic and treatment pathways are relatively straightforward [52], whereas patients in Classes 2 and 3 may require more time for diagnostic evaluations and treatment adjustments [53].

In terms of pain status at discharge, a greater proportion of C1 patients still experienced pain at discharge (60.7%), whereas the “pain-free” proportions for Classes 2 and 3 were 69.7% and 81.8%, respectively. This finding indicates that pain control in C1 patients is more challenging and potentially requires longer-term pain management and follow-up.

The strengths of this study include the use of a comprehensive electronic health record system to extract paediatric pain data and the ability to conduct an in-depth evaluation across multiple dimensions, including pain duration, pain intensity, pain location, inflammation, and functionality. By integrating chief complaints, physical examination findings, and inflammatory factor analyses, this study provides a comprehensive and valid assessment of paediatric pain across multiple dimensions. However, there are certain limitations to consider. First, this was a single-centre, retrospective study that was primarily based in Southwest China and included children from nine provincial administrative regions. As a result, the sample may not fully represent broader populations. The majority of the study sample was Han Chinese, with a lower proportion of ethnic minorities being included. This ethnic composition may limit the generalizability of the findings, as differences in genetic backgrounds, cultural perceptions, and socioeconomic conditions across ethnic groups may influence the perception, expression, and management of pain. Therefore, the findings of this study are primarily applicable to Han Chinese children, and further validation is needed to assess their applicability to other ethnic groups. Additionally, our results have not been validated in populations from other countries with diverse ethnic and geographic backgrounds. Future studies should consider including a more diverse ethnic background to confirm and expand upon the pain phenotypes that were identified in this study and to assess their consistency across different populations. This would help to enhance the external validity and broader applicability of the findings. Furthermore, the retrospective design of this study constrained the analysis of short-term clinical outcomes and did not allow for the exploration of the long-term impact of pain on disease progression and prognosis. Although the patients were newly diagnosed with sJIA and in the acute phase, delays in consultation and diagnosis may have introduced noninflammatory pain components. According to the biopsychosocial model of pain, future studies should consider including psychological and social factors to enhance the understanding of the complex experiences of pain.

## Conclusion

This study highlights the significant heterogeneity of pain occurring among patients with systemic juvenile idiopathic arthritis. By identifying distinct pain phenotypes and their associated factors, we underscore the importance of individualized pain assessment and management in sJIA patients. Personalized approaches that consider patient age, biological markers such as IL-10, and specific pain characteristics can improve clinical outcomes. Furthermore, the integration of multidisciplinary care and holistic medical practices will be essential in addressing the complex needs of children with sJIA.

## Electronic supplementary material

Below is the link to the electronic supplementary material.

Supplementary Material 1: MPLUS Analysis Scripts. Contains MPLUS scripts for analyzing sJIA pain phenotypes.

Supplementary Material 2: sJIA Pain Phenotype Data. Contains the dataset used in the study

## Data Availability

A portion of the data generated or analyzed during this study is included in the article and its supplementary materials. Due to privacy and consent considerations related to research participants, certain data are not publicly available but can be obtained from the corresponding author upon reasonable request.

## Abbreviations

ACR: American College of Rheumatology
sJIA: Systemic Juvenile Idiopathic Arthritis
IL-1, IL-6, IL-10, IL-18: Interleukin-1, 6, 10, 18
TNF-α: Tumor Necrosis Factor-alpha
CRP: C-Reactive Protein
ESR: Erythrocyte Sedimentation Rate
OMERACT: Outcome Measures in Rheumatology
JADAS: Juvenile Arthritis Disease Activity Score
LCA: Latent Class Analysis
AIC: Akaike Information Criterion
BIC: Bayesian Information Criterion
BCH: Bolck-Croon-Hagenaars Method
LTB: Lanza-Tan-Bray
VRS: Verbal Rating Scale
