# Supplementary material for: Latent class analysis identifies distinct pain phenotypes in newly diagnosed systemic juvenile idiopathic arthritis
Source: Arthritis Res Ther. 2025 Mar 31;27:71. doi: 10.1186/s13075-025-03534-7 (PMC11956179; doi:10.1186/s13075-025-03534-7)
Supplement: Supplementary file 1 — Supplementary Material 1: MPLUS Analysis Scripts. Contains MPLUS scripts for analyzing sJIA pain phenotypes. [file 13075_2025_3534_MOESM1_ESM.docx]

File Description: This file contains data used to study pain phenotypes in Systemic Juvenile Idiopathic Arthritis (sJIA). The analysis utilized Latent Class Analysis (LCA), along with the Lanza-Tan-Bray method and the Data Combination Analysis Technique.

**Variable Descriptions**:

1. **time** - Pain duration
2. **sev** - Pain severity
3. **gong** - Functional impairment
4. **count** - Number of painful joints
5. **imf** - Inflammation-related pain
6. **quan** - Extra-articular systemic pain
7. **shou** - Initial symptom as pain
8. **ZK** - Internal transfer
9. **ZY** - Hospitalization days
10. **TT** - Discharge pain situation

The accompanying MPLUS script code for the analysis is provided below:

**1-5 Class Latent Class Analysis：**

DATA:

FILE IS DATA.dat;

VARIABLE:

NAMES ARE time sev gong count imf quan shou ID;

USEVAR ARE time sev gong count imf quan shou;

CATEGORICAL ARE time sev gong count imf quan shou;

CLASSES = C(1);

ANALYSIS:TYPE = MIXTURE;

STARTS = 200 20;

SAVEDATA:FILE IS P1.CSV;

SAVE = CPROB;

OUTPUT:TECH11 TECH14;

FILE IS DATA.dat;

VARIABLE:

NAMES ARE time sev gong count imf quan shou ID;

USEVAR ARE time sev gong count imf quan shou;

CATEGORICAL ARE time sev gong count imf quan shou;

CLASSES = C(2);

ANALYSIS:TYPE = MIXTURE;

STARTS = 200 20;

SAVEDATA:FILE IS P2.CSV;

SAVE = CPROB;

OUTPUT:TECH11 TECH14;

DATA:

FILE IS DATA.dat;

VARIABLE:

NAMES ARE time sev gong count imf quan shou ID;

USEVAR ARE time sev gong count imf quan shou;

CATEGORICAL ARE time sev gong count imf quan shou;

CLASSES = C(3);

ANALYSIS:TYPE = MIXTURE;

STARTS = 200 20;

SAVEDATA:FILE IS P3.CSV;

SAVE = CPROB;

OUTPUT:TECH11 TECH14;

DATA:

FILE IS DATA.dat;

VARIABLE:

NAMES ARE time sev gong count imf quan shou ID;

USEVAR ARE time sev gong count imf quan shou;

CATEGORICAL ARE time sev gong count imf quan shou;

CLASSES = C(4);

ANALYSIS:TYPE = MIXTURE;

STARTS = 200 20;

SAVEDATA:FILE IS P4.CSV;

SAVE = CPROB;

OUTPUT:TECH11 TECH14;

DATA:

FILE IS DATA.dat;

VARIABLE:

NAMES ARE time sev gong count imf quan shou ID;

USEVAR ARE time sev gong count imf quan shou;

CATEGORICAL ARE time sev gong count imf quan shou;

CLASSES = C(5);

ANALYSIS:TYPE = MIXTURE;

STARTS = 200 20;

SAVEDATA:FILE IS P5.CSV;

SAVE = CPROB;

OUTPUT:TECH11 TECH14;

**Short-Term Inpatient Outcomes in sJIA Based on Pain Phenotypes MPLUS Script:**

DATA:

FILE IS DATA.dat;

VARIABLE:

NAMES ARE ID time sev gong count imf quan shou ZK ZY TT;

USEVAR ARE time sev gong count imf quan shou;

CATEGORICAL ARE time sev gong count imf quan shou;

CLASSES = C(3);

AUXILIARY = ZY(BCH);

ANALYSIS:TYPE = MIXTURE;

STARTS = 200 20;

SAVEDATA:FILE IS P3.CSV;

SAVE = CPROB;

OUTPUT:TECH11 TECH14;

DATA:

FILE IS DATA.dat;

VARIABLE:

NAMES ARE ID time sev gong count imf quan shou ZK ZY TT;

USEVAR ARE time sev gong count imf quan shou;

CATEGORICAL ARE time sev gong count imf quan shou;

CLASSES = C(3);

AUXILIARY = TT(DCAT);

ANALYSIS:TYPE = MIXTURE;

STARTS = 200 20;

SAVEDATA:FILE IS P3.CSV;

SAVE = CPROB;

OUTPUT:TECH11 TECH14;

DATA:

FILE IS DATA.dat;

VARIABLE:

NAMES ARE ID time sev gong count imf quan shou ZK ZY TT;

USEVAR ARE time sev gong count imf quan shou;

CATEGORICAL ARE time sev gong count imf quan shou;

CLASSES = C(3);

AUXILIARY = ZK(DCAT);

ANALYSIS:TYPE = MIXTURE;

STARTS = 200 20;

SAVEDATA:FILE IS P3.CSV;

SAVE = CPROB;

OUTPUT:TECH11 TECH14;
